# Interventions for body weight reduction in obese patients during short consultations: an open-label randomized controlled trial in the Japanese primary care setting

**DOI:** 10.1186/s12930-015-0022-7

**Published:** 2015-05-21

**Authors:** Satoshi Kanke, Takumi Kawai, Naomi Takasawa, Yukiko Mashiyama, Atsushi Ishii, Ryuki Kassai

**Affiliations:** Department of Community and Family Medicine, Fukushima Medical University, 1 Hikarigaoka, Fukushima City, Fukushima Prefecture Japan; Department of Internal Medicine, Kashima Hospital, 22-1 Shimokuramochi Nakasawame, Kashima-machi, Iwaki City, Fukushima Prefecture Japan

**Keywords:** Obesity, Weight reduction, Brief intervention, Primary care, Outpatient care, Short consultation

## Abstract

**Background:**

Family physicians should maintain regular contact with obese patients to ensure they effectively reduce their body weight. However, family physicians in Japan have on average only 6 (min) per consultation, and conventional interventions for body weight reduction require a longer consultation or additional manpower. A brief intervention within the limited consultation time available is therefore needed. Here we investigated the effectiveness of a brief weight reduction intervention for obese patients and the related factors for reducing body weight during routine consultations in the primary care setting.

**Method:**

We conducted an open-label randomized controlled trial at a family medicine clinic in Fukushima, Japan from January 2010 to June 2011. Patients aged 30 to 69 years with body mass index ≥25 who were diagnosed with hypertension, dyslipidemia, and/or type 2 diabetes mellitus were randomly assigned to the intervention or control group. At every consultation, body weight in the intervention group was measured by a family physician who provided weight reduction advice in addition to usual care. The primary outcome was body weight change at 1-year follow up. Analysis was done by intention to treat.

**Result:**

We randomly assigned 29 participants to the intervention group and 21 to the control group. Forty participants (80 %) remained in the trial until the 1-year follow up. At follow up, the median body weight change from baseline was not significantly different between the groups (p = 0.68), at −0.8 (interquartile range [IQR] −2.5 to 1.0) kg in the intervention group and 0.2 (IQR −2.4 to 0.8) kg in the control group.

**Conclusion:**

We devised an intervention method for physicians to measure body weight and advise on weight reduction during routine consultations. In our setting, this method did not extend the consultation time, but also had no significant additional effects on body weight reduction in moderately obese patients.

**Trial registration:**

This trial is registered with the UMIN Clinical Trial Registry (UMIN000002967).

## Background

The number of patients with hypertension, dyslipidemia, or type 2 diabetes mellitus has increased in the last few decades in Japan [1]. From 2002 to 2011, the number of patients with hypertension increased from 7 million to 9 million, those with dyslipidemia from 1.4 million to 1.9 million, and those with type 2 diabetes from 2.3 million to 2.7 million [1]. All three of these diseases are related to obesity [2, 3]. In 2008, more than 10 % of the world’s adult population was obese according to the World Health Organization’s definition of a body mass index (BMI) ≥30 kg/m^2^ [4], although only 3 % of the Japanese population in 2011 conformed to this definition of obesity [5]. As the incidence rates of obesity-related diseases in Japan have been increasing, an international expert panel proposed a lower BMI cut-off for the Japanese population [6]. The current definition of obesity for Japanese is BMI ≥25 kg/m^2^ [7]. According to this definition, 30 % of Japanese adult men and 21 % of Japanese adult women were reported to be obese in 2011 [5]. Developing an effective intervention for reducing the body weight of obese Japanese patients has the potential to improve the management of obesity-related diseases.

Although the guidelines for managing overweight and obesity recommend advising patients with obesity-related diseases to lose weight [8], the weight reduction approach of healthcare providers remains inadequate [9]. Primary care physicians meet many obese patients with obesity-related diseases [10] and should maintain regular contact with these patients to ensure they reduce their body weight as necessary. Epstein and Ogden revealed conflicting points of view between primary care physicians and obese patients. Obese patients regarded obesity as a medical problem that should be managed by the physicians. In contrast, physicians consider obesity management to be primarily the patient’s responsibility. One of the reasons for this discord is the lack of effective patient interventions in the primary care setting [11]. In the primary care setting, physicians need a brief and easy-to-perform intervention method for encouraging obese patients to lose weight. Because physicians manage on average three problems during each short consultation [12], it is too difficult to provide the proven intervention methods established in other studies in a real-world clinical setting. In previous studies conducted in the primary care setting outside of Japan, patient body weight has been effectively reduced through lifestyle counseling provided by medical assistants, nurses, or dietitians [13–15], as well as by Internet-based intervention programs [16, 17]. In Japan, providing these interventions in the primary care setting is difficult because very few dietitians work for primary care clinics and clinic nurses do not have sufficient experience in lifestyle counseling. In fact, most lifestyle counseling is given by Japanese primary care physicians during regular patient visits [18]. In other studies, physicians have provided tailored intervention to obese patients during 15 to 20-min long consultations [19, 20]; however, in Japan, primary care consultations last for around 6 min [21], meaning that primary care physicians cannot practically provide any effective counseling-based interventions due to time constraints. In the Japanese primary care setting, physicians need simple and easy-to-perform methods of intervention that are suitable for use in routine brief consultations.

In our clinical experience, some of our obese patients, whose body weight was checked by the physician at every consultation, managed to reduce their body weight. Based on this success, we focused on body weight monitoring as an innovative method for promoting body weight reduction in the primary care setting. In general, weight reduction strategies consist of the following five approaches: dietary intervention, physical activity, behavioral treatment, pharmacotherapy, and surgical therapy [3]. Many studies in the primary care setting have reported the effects of dietary intervention, physical activity, and pharmacotherapy on body weight reduction in obese patients [13–15]. However, weight monitoring is a component of behavioral treatment, and in the area of behavioral treatment, most studies on body weight reduction have applied an intensive approach in an academic setting; few studies have reported the effects of behavioral treatment on body weight reduction in the primary care setting [22].

Against this background, we hypothesized that if physicians involved in outpatient care measure patient body weight and advise on weight reduction at every consultation, this approach might reduce the body weight of obese patients since it can be performed quickly and easily. There are several important points regarding the approach taken in this study. First, obese patients should monitor their weight with their physician since weight monitoring enables obese patients to recognize their actual condition. We planned weight monitoring with a physician at every consultation to ensure a sense of urgency and motivation for lifestyle change. Second, physicians should ask patients about their lifestyles based on the measured body weight and provide specific advice on aspects that are difficult to improve. Third, an intervention method for lifestyle change is needed to improve doctor-patient relationships [23, 24]. Fourth, we planned to establish a trust relationship by requesting the physician to advise on the intervention method. The objective of this study, therefore, was to devise a brief and easy-to-perform intervention method for body weight reduction in the Japanese primary care setting.

## Methods

### Design and participants

**Fig. 1:**
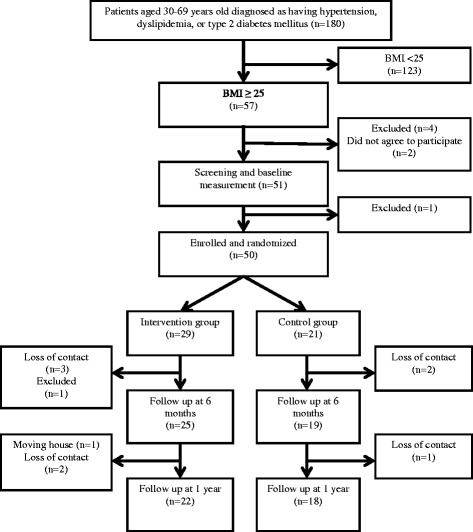
Participant flow chart

We planned an open-label randomized control study at a family medicine clinic (Date City, Fukushima Prefecture, Japan) attended by four family physicians. Date city is located 250 km north of Tokyo, its population was about 66,000 people in 2010, and main industry of the city is agriculture. From January to June 2010, we checked the medical records of adult patients aged 30–69 years who visited the family medicine department for routine checkups for hypertension, dyslipidemia, and/or type 2 diabetes mellitus. A total of 180 patients were matched in January 2010. From the pool, we recruited 57 patients with a BMI ≥25 kg/m^2^ (moderately to severely obese) to participate in this study. We excluded patients with a history of cancer or psychological disease, or those prescribed hormone therapy because these factors are known to affect body weight [25–27]. We informed all participants of the aim and content of the study, and obtained their written consent to participate. After being informed of this research project, 51 patients agreed to participate. One participant with a history of cancer was excluded. At baseline measurement, 50 participants (18 women, 36 %; 32 men, 64 %) were enrolled. We randomized 29 participants to the intervention group and 21 participants to the control group. In total, 44 participants (88 %) were assessed for body weight changes at 6 months, and 40 participants (80 %) remained in the study until the 1-year follow up (Fig. 1).

### Baseline data and measurement

After the participants agreed to participate, their physician collected the following baseline data: age, sex, history of hypertension (yes or no), history of dyslipidemia (yes or no), history of type 2 diabetes (yes or no), educational background (junior high school, high school, technical college, or university), history of smoking (never smoker, former smoker, or current smoker), history of alcohol consumption (never drinker, <once a week, <3 times per week, <6 times per week, or daily), and frequency of body weight self-monitoring (<once a month, <once a week, several times per week, or daily).

Nurses, who were blinded to the group assignment of each patient, measured participant height, body weight, abdominal circumference, and blood pressure. After defecating and urinating, participants removed their shoes and wore as light clothing as possible for height and body weight measurement on a digital scale. Abdominal circumference was measured at the umbilical level with a tape measure in the standing position and at end-expiratory pressure [28], and blood pressure was measured in the sitting position with an automated sphygmomanometer after a few minutes rest. We checked these data and the participants’ medical records to ensure the participants met the criteria for metabolic syndrome (Japanese criteria and the National Cholesterol Education Program-Adult Treatment Panel III criteria) [28, 29].

### Randomization

Participants were assigned original identification numbers, which were sent to a co-researcher (AI) working at another hospital who randomized these numbers into two groups (intervention group and control group) by means of a table of random numbers. The randomization results were sent to the chief researcher (SK) who informed the participants. Figure 1 shows the participant flow throughout the study.

### Intervention

In the intervention group, at the first consultation after randomization, participants were informed about their ideal body weight, weight reduction target (5 % of current weight), and the positive effect of weight reduction for their current diseases by the physician, who provided them with an information leaflet [30]. Participants were also informed that a physician would check their body weight and provide specific advice on weight reduction at every regular consultation. Thereafter, the participants received care for their current diseases based on the Japanese guidelines for hypertension, dyslipidemia, and type 2 diabetes [31–33] every 1 or 2 months. A physician measured body weight after defecation and micturition using an analogue scale at every consultation and provided advice in relation to the changes of measured body weight at each consultation. In addition, at every consultation, the physician was required to specifically question the patient on eating and exercise, to reconfirm the patient’s weight target, and to provide advice focusing on each participant’s difficulties in lifestyle change. We requested all physicians to perform this intervention, even if the consultation duration exceeded the usual appointment time (Table 1).

**Table 1:** Consultation content

	Intervention group	Control group
First consultation after randomization	Explanations were given on the following:	Explanations were given on the following:
	*Ideal body weight (body mass index, 22 kg/m* ^*2*^ *) and weight reduction target of each participant (5 % body weight).*	*Ideal body weight (body mass index, 22 kg/m* ^*2*^ *) and weight reduction target of each participant (5 % body weight).*
	*The positive effect of weight reduction for the participant’s present disease.*	*The positive effect of weight reduction for the participant’s present disease.*
	*A physician measured the participant’s body weight and provided specific advice on weight reduction at every routine consultation.*	
Every subsequent routine consultation	Routine consultations were performed every 1 or 2 months for the participant’s present disease based on the guidelines for the disease.	Routine consultations were performed every 1 or 2 months for the participant’s present disease based on the guidelines for the disease.
	Body weight was measured.	
	The physician questioned the patient on key lifestyle factors for weight reduction (i.e., eating, exercising, and weight monitoring)	
	The physician provided information on the following standard lifestyle changes for obese people:	
	*Calorie intake (reduce calorie intake to 25 kcal/kg ideal body weight/day).*	
	*Eat a well-balanced diet (calorie balance: protein, 10-15 %; fat, 20-25 %, and carbohydrate, 60 %).*	
	*Exercise for 20-30 min at least three times per week.*	
	The physician provided advice focusing on weight reduction adjusted to each participant’s circumstances and lifestyle.	

In the control group, at the first consultation after randomization, participants were informed of their ideal body weight, weight reduction target, and positive effect of weight reduction, as in the intervention group. Thereafter, participants received care for their current diseases based on the Japanese guidelines every 1 or 2 months. However, the physician was not required to measure body weight or discuss body weight reduction at every consultation.

### Outcomes

The primary outcome of this study was change in body weight at 1-year follow up. Secondary outcomes were changes in body weight at 6-month follow up, and changes in abdominal circumference and blood pressure at the 6-month and 1-year follow ups. Nurses blinded to the group assignment of each patient measured body weight, abdominal circumference, and blood pressure at 6 months and 1 year. The nurses then asked the participants about their frequency of weight self-monitoring at the 1-year follow up using the same classification as that used at baseline. Physicians recorded the length (in minutes and seconds) of each consultation and calculated the mean length for each patient after all participants had completed the 1-year follow up.

### Sample size calculation

We selected a mean weight difference of 2 kg after 1 year as clinically significant and assumed a standard deviation of weight change of 2 kg, in accordance with a previous study [34]. The present study was designed to have an 80 % power to detect a weight change of 2 kg in both groups. For this purpose, at least 17 participants were needed in each group. We considered p <0.05 as statistically significant.

### Blinding

We informed the participants and the physicians, but not the nurses, of the randomization results at the first consultation after randomization. No measurement data were given to the physicians. Analysis was performed after all data had been collected.

### Statistical analyses

Analysis was based on the intention-to-treat principle. The categorical variables of baseline data were converted into binary categories, education background (<high school or ≥ high school), history of smoking (nonsmoker or currently smoker), history of drinking alcohol (<once a week or ≥ once a week), and frequency of body weight self-monitoring (<once a week or ≥ once a week). We analyzed baseline data between the two participant groups using the Mann–Whitney *U* test and Chi-squared test. The main outcome and secondary outcomes were analyzed using the Mann–Whitney *U* test. Statistical analysis was performed using SPSS Statistics (version 17.0; SPSS Inc, Chicago, IL).

### Ancillary analyses

Further analyses were conducted on the associations between body weight change and consultation factors. Data of 40 participants, regardless of their randomization group, who completed the 1-year follow up were used. We focused on the consultation factors, which included the number of consultations over 1 year, total length of consultations over the year, mean length of each consultation, and number of physicians who saw participants in the clinic over the year, in order to calculate the total consultation length over the year and the mean length of each consultation. The number of physicians who saw participants was obtained from the medical records. The associations of these factors with the change in body weight over the 1-year period were analyzed using Spearman’s rank correlation coefficient.

In another ancillary analysis, we focused on the weight self-monitoring factors, which included their frequency and change over the 1-year period. From the self-reported weight self-monitoring frequency at baseline and at the 1-year follow up, we divided the participants into the following two groups based on change in weight self-monitoring frequency: those with a decreased frequency (decreased group), and those with an increased or unchanged frequency (non-decreased group). We then analyzed the relationship between amount of body weight change at 1 year and the frequency, or change in the frequency, of weight self-monitoring over the year. Data were analyzed using the Kruskal-Wallis test and the Mann–Whitney *U* test.

This study was approved by the Ethics Board of Fukushima Medical University (No. 905) and is registered in the UMIN Clinical Trial Registry (UMIN 000002967).

## Results

For participants in both groups, the mean age (SD) was 54.8 (6.7) years, mean body weight (SD) was 74.1 (9.6) kg, and mean BMI (SD) was 28.1 (2.1) kg/m^2^. At baseline, no significant differences were evident between the groups (Table 2). In one year follow-up period, 12 participants changed regular prescription drugs (Table 3). The median number of consultations (interquartile range [IQR]) was 8 (7 to 10) in the intervention group and 10 (9 to 11) in the control group. The median consultation length (IQR) for each patient over the 1-year period was 59.1 (51.4 to 71.1) min in the intervention group and 79.7 (64.8 to 97.8) min in the control group. The median length (IQR) of each consultation was 7.0 (6.3 to 8) min in the intervention group and 8.0 (6.4 to 9.8) min in the control group. The median number of doctors (IQR) who saw each participant over the 1-year period was 2 (1 to 3) in the intervention group and 2 (1 to 3) in the control group. The number of consultations (p = 0.01) and the total consultation length (p = 0.002) were significantly different in both groups. The length of each consultation was not significantly different (p = 0.13) (Table 4).

**Table 2:** Baseline demographic and clinical characteristics of participants

	*n (%)* or Median (interquartile range)				
	Intervention		Control		*p* value
	n = 29		n = 21		
Age (years)	56	(38 to 65)	55	(42 to 63)	0.94
Sex					0.39
Female	*9*	*(31)*	*9*	*(43)*	
Male	*20*	*(69)*	*12*	*(57)*	
Weight (kg)	71.8	(67.3 to 82.4)	74.1	(68.1 to 77.4)	0.84
Height (cm)	164.5	(156.5 to 168.3)	162.5	(154.5 to 168.1)	0.79
BMI (kg/m^2^)	27.6	(26.4 to 29.5)	27.6	(26.9 to 28.4)	0.78
Abdominal circumference (cm)	94.0	(91.8 to 98.0)	95.0	(92.0 to 97.5)	0.93
Blood pressure					
Systolic blood pressure (mmHg)	130	(120 to 140)	132	(120 to 138)	0.85
Diastolic blood pressure (mmHg)	82	(72 to 86)	78	(71 to 86)	0.88
Serum lipid profile					
Low-density lipoprotein (mg/dl)	128	(101 to 147)	126	(103 to 148)	0.86
High-density lipoprotein (mg/dl)	56	(45 to 61)	53	(45 to 62)	0.85
Triglyceride (mg/dl)	117	(88 to 171)	109	(88 to 126)	0.52
Fasting blood sugar (mg/dl)	100	(95 to 109)	102	(95 to 117)	0.42
Hemoglobin A1c (%)	5.5	(5.2 to 5.9)	5.7	(5.3 to 6.2)	0.15
Regular medication					
Antihypertensive drug	*24*	*(82)*	*15*	*(71)*	0.49
Lipid-lowering drug	*9*	*(31)*	*7*	*(33)*	0.86
Anti-diabetic drug	*0*	*(0)*	*5*	*(24)*	0.01
Medical history					
Hypertension	*25*	*(86)*	*17*	*(81)*	0.71
Dyslipidemia	*11*	*(38)*	*8*	*(38)*	0.99
Type 2 diabetes mellitus	*2*	*(7)*	*6*	*(29)*	0.06
Metabolic syndrome criteria					
Japanese criteria	*15*	*(52)*	*6*	*(29)*	0.10
NCEP-ATP III criteria	*15*	*(52)*	*7*	*(33)*	0.20
Educational background					0.48
Under high school	*7*	*(24)*	*7*	*(33)*	
High school and above	*22*	*(76)*	*14*	*(67)*	
Smoking					0.87
Non-smoker	*24*	*(83)*	*17*	*(81)*	
Currently smoker	*5*	*(17)*	*4*	*(19)*	
Alcohol drinking					0.53
Under once a week	*14*	*(48)*	*12*	*(57)*	
Once a week or more	*15*	*(52)*	*9*	*(43)*	
Weight self monitoring frequency^a^					0.73
Under once a week	*14*	*(48)*	*11*	*(52)*	
Once a week or more	*14*	*(48)*	*9*	*(43)*	
Home blood pressure monitoring					0.19
Regularly	*24*	*(83)*	*14*	*(67)*	
Not regularly	*5*	*(17)*	*6*	*(33)*	

We compared groups with the Mann–Whitney *U* test or Chi-squared test

BMI, body mass index; NCEP-ATP, Cholesterol Education Program-Adult Treatment Panel

^a^Data were missing for one patient in the intervention group and one patient in the control group

**Table 3:** Regular prescription drugs change between baseline and the 1-year follow up

	Number of participants, n (%)			
	Intervention group		Control group	
	n = 22		n = 18	
Antihypertensive drugs				
Increase	1	(5)	5	(28)
Stable	21	(95)	13	(72)
Decrease	0		0	
Lipid-lowering drug				
Increase	3	(14)	1	(6)
Stable	19	(86)	17	(94)
Decrease	0		0	
Anti-diabetic				
Increase	0		1	(6)
Stable	22	(100)	16	(88)
Decrease	0		1	(6)

**Table 4:** Consultation factors in the intervention and control groups

	Median (interquartile range)				
	Intervention group		Control group		*p* value
	n = 22		n = 18		
Number of consultations in one year	8	(7 to 10)	10	(9 to 11)	0.01*
Total consultation duration in one year (min)	59.1	(51.4 to 71.1)	79.7	(64.8 to 97.8)	0.002*
Average duration of consultation (min)	7.0	(6.3 to 8.0)	8.0	(6.3 to 9.9)	0.13

We compared groups with the Mann–Whitney *U* test

*Significantly different (p <0.05) between the groups

**Fig. 2:**
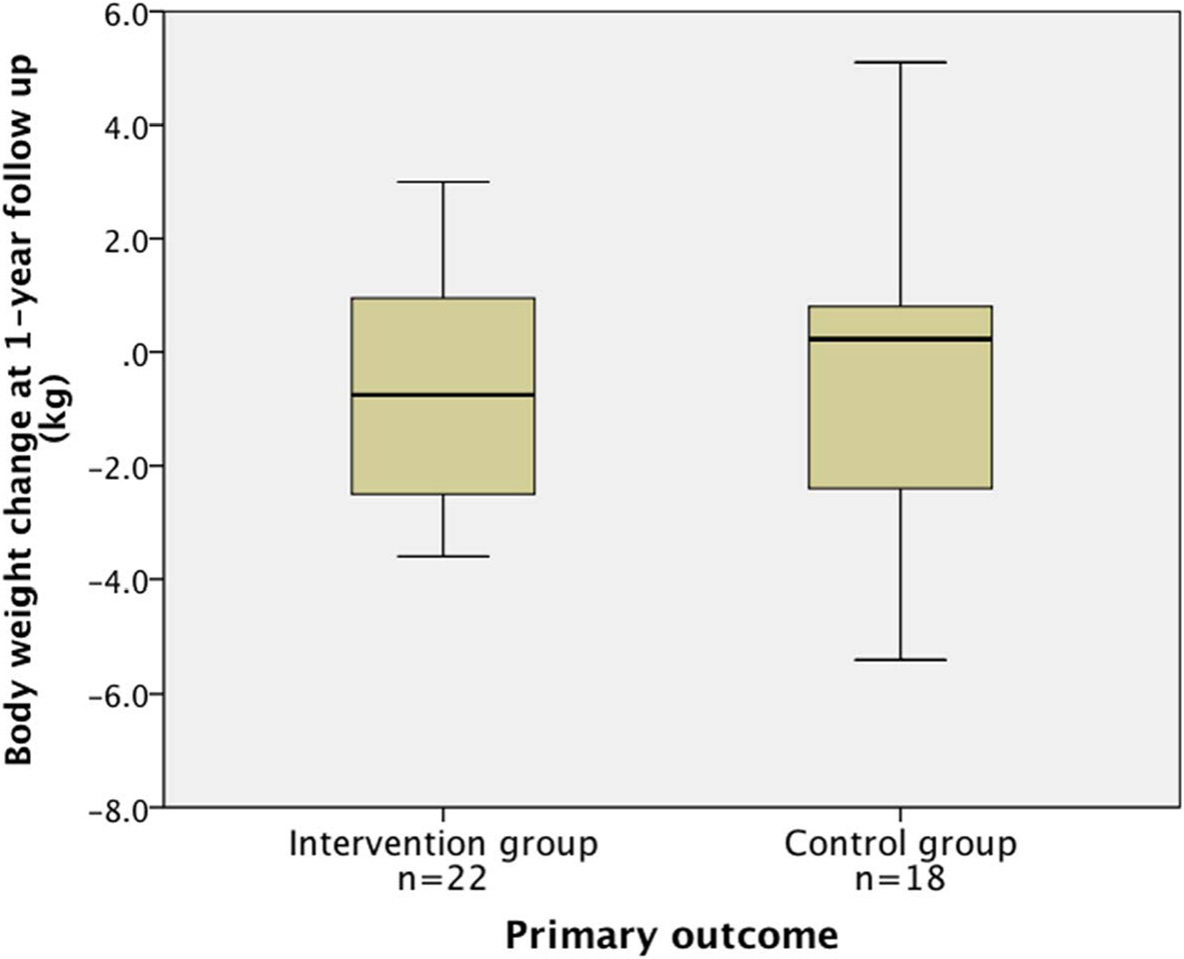
Box plots of body weight change at the 1-year follow up

Intention-to-treat analysis revealed that at 1 year, 15 participants in the intervention group (68 % of the group) and 9 participants in control group (50 % of the group) had decreased their body weight from baseline values. The median (IQR) change in body weight from baseline was −0.8 (−2.5 to 1.0) kg in the intervention group and 0.2 (−2.4 to 0.8) kg in the control group. There was no significant difference in this primary outcome (p = 0.68) observed between the groups (Fig. 2).

**Fig. 3:**
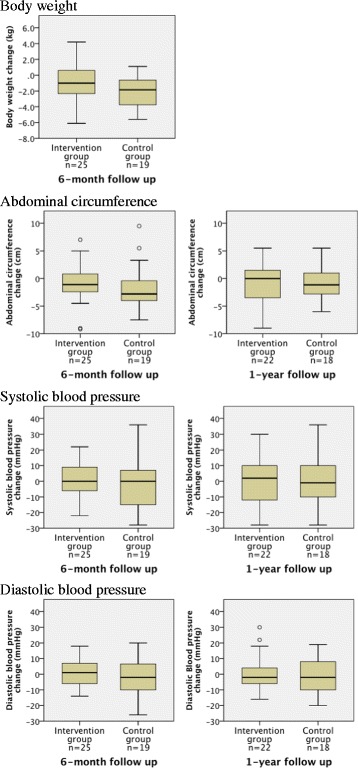
Box plots of secondary outcomes

No significant differences in secondary outcomes were noted between the groups; that is, the median (IQR) changes in the intervention and control groups at the 1-year follow up were 0.0 (−3.5 to 1.5) cm and −1.2 (−2.8 to 1.0) cm for abdominal circumference, 2 (−12 to 10) mmHg and −1 (−10 to 7) mmHg for systolic blood pressure, and −2 (−6 to 4) mmHg and −2 (−10 to 8) mmHg for diastolic blood pressure, respectively. The median (IQR) changes in the intervention and control groups at 6 months were −1.0 (−2.3 to 0.6) kg and −1.9 (−3.8 to 0.6) kg for body weight, −1.1 (−2.4 to 0.9) cm and −2.8 (−4.0 to −0.4) cm for abdominal circumference, 0 (−6 to 9) mmHg and 0 (−15 to 7) mmHg for systolic blood pressure, and −1 (−6 to 7) mmHg and −2 (−10 to 7) mmHg for diastolic blood pressure, respectively (Fig. 3). Again, no significant differences were observed between the groups.

### Ancillary analyses

In analysis of consultation factors, the mean number of consultations (SD) was 9.1 (2.1), mean total consultation length (SD) over 1 year was 70.6 (24.9) min, mean length of each consultation (SD) was 7.8 (2.2) min, and mean number of physicians who saw each participant (SD) was 2.1 (1.0). The correlation coefficient was 0.16 (p = 0.31) for body weight change and the number of consultations, 0.32 (p = 0.05) for body weight change and the total consultation length, 0.30 (p = 0.06) for body weight change and length of each consultation, and −0.01 (p = 0.93) for body weight change and the number of physicians who saw each participant. A weak positive correlation was seen between body weight change and total consultation length over the year.

**Fig. 4:**
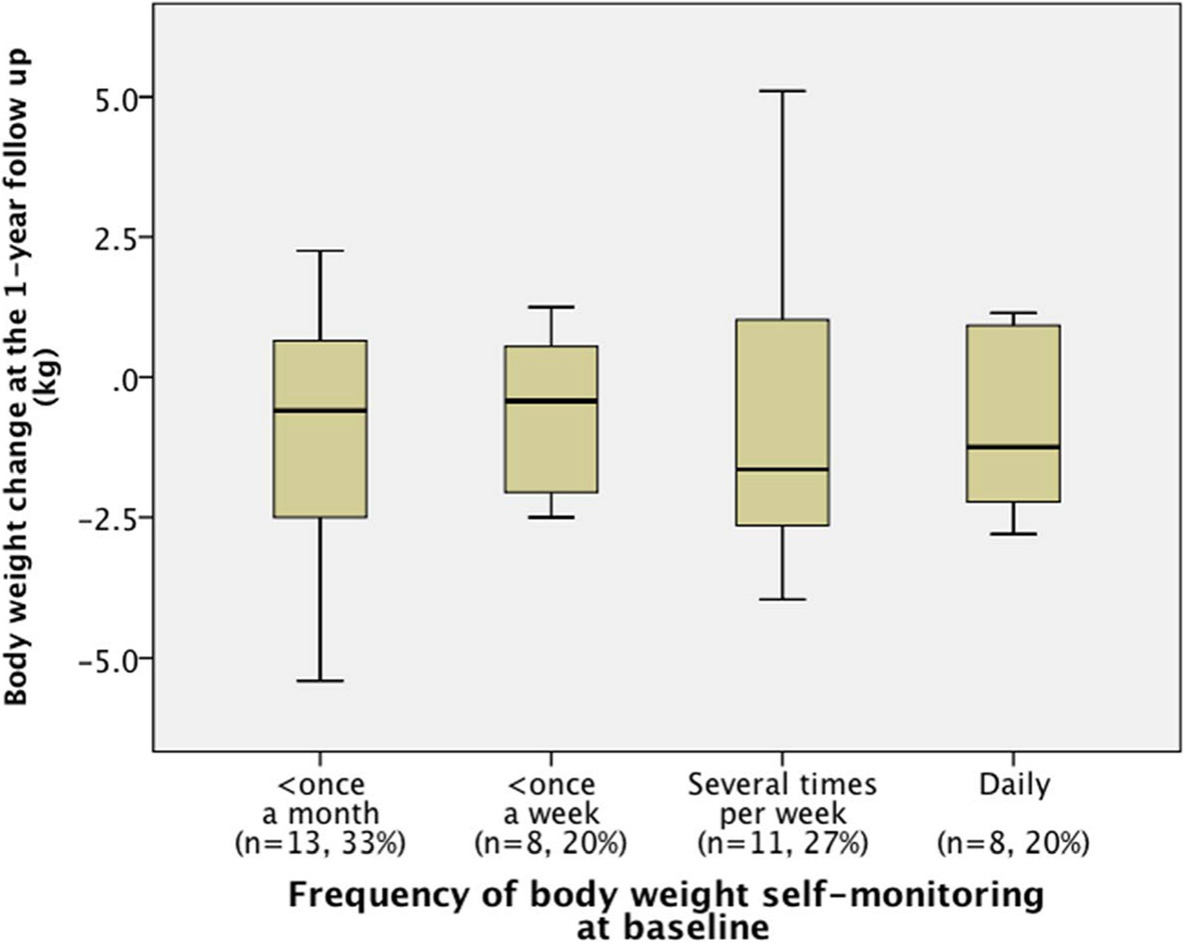
Box plots of body weight change at the 1-year follow up in relation to the frequency of self-monitoring at baseline

**Fig. 5:**
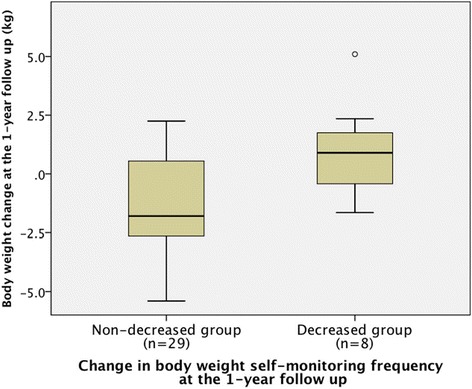
Box plots of body weight change in 1-year follow up by body weight self-monitoring frequency

In another ancillary analysis about body weight self-monitoring, at baseline, weight self-monitoring was performed less than once a month by 13 (33 %) participants, less than once a week by 8 (20 %) participants, several times per week by 11 (28 %) participants, and daily by 8 (20 %) participants. The median (IQR) of body weight change at the 1-year follow up was −0.6 (−2.7 to 0.7) kg in participants self-monitoring less than once a month, −0.4 (−2.2 to 0.9) kg in those self-monitoring less than once a week, −1.7 (−2.9 to 1.1) kg in those self-monitoring several times per week, and −1.3 (−2.7 to 1.1) kg in those self-monitoring daily (Fig. 4). Analysis using the Kruskal-Wallis test showed no significant differences (p = 0.86). We calculated the change in the frequency of weight self-monitoring from baseline to the 1-year follow up (Table 5). Eight participants (21 %) had decreased their frequency of weight self-monitoring, 20 (54 %) showed no change, and 9 (24 %) had increased their frequency at the 1-year follow up. We analyzed body weight change over the 1-year period between the decreased group (n = 8) and the non-decreased group (n = 29). The median (IQR) changes in body weight between baseline and the 1-year follow up were −1.8 (−2.7 to 0.6) kg in the non-decreased group and 0.9 (−0.4 to 1.8) kg in the decreased group, a significant difference (p = 0.009) between the groups (Fig. 5).

**Table 5:** Comparison of body weight self-monitoring frequency at baseline and at the 1-year follow up

		Number of participants, n (%)									
		Frequency of body weight self-monitoring at the 1-year follow up									
		<Once a month		<Once a week		>Once a week		Daily		Total	
Frequency at Baseline	<Once a month	6	(16)	3	(8)	1	(3)	1	(3)	11	(29)
	<Once a week	3	(8)	3	(8)	2	(5)	0	(0)	8	(22)
	>Once a week	2	(5)	2	(5)	5	(14)	2	(5)	11	(29)
	Daily	0	(0)	1	(3)	0	(0)	6	(16)	7	(19)
	Total	11	(30)	9	(24)	8	(22)	9	(24)	37	(100)

It should be noted that the study area was affected by the Great East Japan Earthquake in March 2011, which occurred during the study period. In the aftermath, both water and gasoline were in short supply, which forced some participants to evacuate to community halls for several days. The 1-year follow up for 11 participants of the intervention group (50 % of the group) and 9 in the control group (50 % of the group) (total 20; 50 %) was done after the earthquake, and we analyzed the effect of the earthquake on body weight change using the Mann–Whitney *U* test (Table 6). No significant differences were observed in body weight change between the participants who attended the 1-year follow up before the earthquake and those who attended after it (p = 0.95).

**Table 6:** Comparison of weight change at the 1-year follow up between before and after the Great East Japan Earthquake

				Total
		Before the earthquake	After the earthquake	
Intervention group	Number of participants	9	9	18
	Weight change (kg)^a^	−0.4 kg	−0.7 kg	−0.5 kg
Control group	Number of participants	11	11	22
	Weight change (kg)^a^	−1.0 kg	−0.6 kg	−0.8 kg
Total	Number of participants	20	20	40
	Weight change (kg)^a^	−0.7 kg	−0.6 kg	−0.7 kg

^a^Mean weight change between baseline and the 1-year follow up

## Discussion

We planned this study to reveal the effects on obese Japanese patients of adding a brief intervention for body weight reduction to routine consultations with a physician. We accomplish the randomized controlled trial at a family medicine clinic in the primary care setting. Given the lower incidence rate of severe obesity in Japan than in developed Western countries [1, 4], we need an intervention method that not only addresses severe obesity, but also overweight and moderate obesity. In this study, only 8 participants (16 %) were severely obese (BMI ≥30, Japanese criteria), and the average BMI was 28.1, which is classed as moderately obese.

Our results revealed no significant differences in consultation length between the intervention and control groups. This intervention method was performed briefly as planned and proved suitable for short routine consultations. It is noteworthy that the present intervention method is feasible for short routine consultations in the real-world clinical setting.

The total consultation length was unexpectedly longer in the control group than in the intervention group. The consultation length for the control group might have been longer because the control group had a higher ratio of participants who gained body weight. A possible explanation for this finding is that physicians may have extended the duration of each consultation for patients who gained weight and may have kept consultations short for patients who maintained or lost weight. Alternatively, physicians may have extended the consultation time in general to measure body weight and provide advice to patients in the intervention group within the short consultation time allotted and in doing so might have unintentionally overlooked other aspects of care while being preoccupied with the measurement, keeping the consultation time shorter for the intervention group overall.

Although we planned a brief and easy-to-perform intervention method for body weight reduction, no significant additional effects on usual care were observed. On the other hand, the result does not deny effect of all other simple low cost interventions. Brief advice provided by physicians has been effective for quitting smoking [35] in the primary care setting, and simple intervention methods used by primary care physicians to encourage patients to quit smoking have been established [36]. Weight reduction is more complex than smoking cessation in two regards. First, smoking is not essential to survival. Physicians, therefore, can provide clear advice to stop smoking. On the other hand, patients cannot simply stop eating. Physicians can, however, advise on how to eat. Quitting a behavior can be a simpler approach than finding an appropriate way to adjust a behavior. Second, is the nature of underlying medical conditions. Since smoking is a behavioral risk factor for health, physicians can focus their advice directly on smoking behavior. On the other hand, obesity is a biomedical condition resulting from the interactions between social, behavioral, cultural, physiological, metabolic, and genetic factors [37]. To successfully reduce weight, obese patients need to change their eating, exercising, working, and other behaviors. Consequently, physicians need to intervene in multiple factors for weight reduction when dealing with obese patients.

Multiple categories of intervention for body weight reduction are available. The intervention method used in this study was based on a behavioral approach, for which we attached high value to an easy-to-perform method. Previous studies have shown that intervention methods involving multiple approaches for weight reduction are more effective than those with a single approach [14, 38, 39]. Thus, to develop an effective intervention method for reducing body weight during short, primary care consultations, it may be useful to encompass multiple brief and easy-to-perform approaches suited to short consultations.

The approach of our clinic may affect outcomes. As effective doctor-patient communication can improve patient health outcomes [24], family medicine trainees need to improve their skills in doctor-patient communication during residency training. The family medicine clinic, which served as a teaching clinic in the family medicine residency program, had four family physicians. One of the physicians was a faculty member and the other three were senior trainees of family medicine. In this case, more experienced family physicians could have made the difference in the intervention method.

As another consideration for devising a brief and easy-to-perform intervention method, we noted weight self-monitoring. In a previous study, Butryn and colleagues showed that more frequent body weight self-monitoring is related to body weight maintenance in patients who had lost weight [40]. Some researchers have proposed that weight self-monitoring increases obese patients’ awareness of their weight and results in them modifying their eating and exercise behavior [41]. The result of ancillary analysis revealed the relationship between self-monitoring frequency at baseline and body weight reduction, as well as that between change in body weight self-monitoring frequency and body weight reduction. We, therefore, consider it worthwhile to explore this relationship further in a future prospective study.

This study has several limitations. First, it was conducted at a single family medicine clinic, where it was difficult to analyze subjects by sex, age, and other factors due to the number of eligible patients. If the number of participants had been larger, we might detect modest weight reduction. And if the observation period had been longer, we might detect long-term weight change. In real primary care setting, even if effect sizes are small, simple low-cost intervention methods can be used without too much difficulty for years and, therefore, they are of benefit to the patients. A cluster randomized controlled multicenter design is a possible solution for assembling more participants and acquiring more evidence with greater power. The second limitation is that this study was conducted in the Japanese clinical setting, which differs from that in Western countries in terms of shorter and more frequent consultations. The third limitation is that the intervention method was affected by doctor-patient communication as we did not strictly standardize the weight reduction advice provided by the physicians. This might have resulted in inter-physician differences in the intervention, which were not measured. If we had evaluated doctor-patient communication, we would have been able to take variables of patients’ viewpoint. The fourth limitation is that this study did not have data about diet and exercise. We could not analyze about food choice and other actions of the participants. A final limitation is the effects of the Great East Japan Earthquake. In particular, we could not eliminate the effects of the earthquake because of the small study population.

## Conclusions

We studied the intervention method used by physicians to measure body weight and advise patients on weight reduction during routine consultations. In our setting, this method did not extend the consultation duration, but also had no significant effects on body weight reduction in moderately obese patients. Therefore, additional studies on simple and easy-to-perform intervention methods are needed. Taken together, the present findings revealed a potential research question on the relationship between weight self-monitoring and body weight reduction in the primary care setting.
